# NSCLC cells demonstrate differential mode of cell death in response to the combined treatment of radiation and a DNA-PKcs inhibitor

**DOI:** 10.18632/oncotarget.2975

**Published:** 2015-02-28

**Authors:** Lan Yu, Zeng-Fu Shang, Feng-Ming Hsu, Zhang Zhang, Vasu Tumati, Yu-Fen Lin, Benjamin P.C. Chen, Debabrata Saha

**Affiliations:** ^1^ Department of Radiation Oncology, Simmons Comprehensive Cancer Center at UT Southwestern Medical Center, Dallas, TX, USA; ^2^ School of Radiation Medicine and Protection, Medical College of Soochow University, Suzhou Industrial Park, China; ^3^ Department of Urology and Department of Oncology, National Taiwan University Hospital, National Taiwan University College of Medicine, Taipei, Taiwan; ^4^ Collaborative Innovation Center of Radiation Medicine of Jiangsu Higher Education Institutions, Suzhou Industrial Park, China

**Keywords:** Radiosensitizer, Mitotic Catastrophe, Apoptosis, Autophagy, Senescence

## Abstract

The current standard of care for lung cancer consists of concurrent chemotherapy and radiation. Several studies have shown that the DNA-PKcs inhibitor NU7441 is a highly potent radiosensitizer, however, the mechanism of NU7441's anti-proliferation effect has not been fully elucidated. In this study, the combined effect of NU7441 and ionizing radiation (IR) in a panel of non-small cell lung cancer cell lines (A549, H460 and H1299) has been investigated. We found that NU7441 significantly enhances the effect of IR in all cell lines. The notable findings in response to this combined treatment are (i) prolonged delay in IR-induced DNA DSB repair, (ii) induced robust G2/M checkpoint, (iii) increased aberrant mitosis followed by mitotic catastrophe specifically in H1299, (iv) dramatically induced autophagy in A549 and (v) IR-induced senescence specifically in H460. H1299 cells show greater G2 checkpoint adaptation after combined treatment, which can be attributed to higher expression level of Plk1 compared to A549 and H460. The enhanced autophagy after NU7441 treatment in A549 is possibly due to the higher endogenous expression of pS6K compared to H1299 and H460 cells. In conclusion, choice of cell death pathway is dependent on the mutation status and other genetic factors of the cells treated.

## INTRODUCTION

Non-small cell lung cancer (NSCLC) is one of the most commonly diagnosed cancers and the leading cause of cancer-related death worldwide [1]. Current standard of care consists of concurrent chemotherapy and radiation but overall survival rates remain dismal in all but earliest stages of treatment [2, 3]. Therefore, identifying newer targets and developing cytotoxic drugs to increase radiosensitivity is an essential strategy in the treatment of NSCLC.

DNA-PKcs is an essential component in the non-homologous end-joining (NHEJ) pathway of double-stranded DNA break (DSB) repair [4]. In response to DNA DSBs, DNA-PKcs is rapidly recruited to damage sites by the Ku70/Ku80 heterodimer and phosphorylated at Thr2609 and Ser2056 clusters by ATM and itself, respectively [5, 6]. The recruitment and phosphorylation of DNA-PKcs contributes to processing and direct ligation of broken DNA ends. In addition to its role in DNA DSB repair, DNA-PKcs also participates in DNA DSB-induced G2/M cell cycle checkpoint regulation and apoptosis [7, 8]. Several studies have demonstrated the elevation of mRNA and protein levels of DNA-PKcs in NSCLC [9–11], and, furthermore, the elevation of DNA-PKcs is correlated with radioresistance in some advanced stage cancers [11]. Therefore, targeting of DNA-PKcs is an attractive approach to enhance radiosensitivity in NSCLC. Based upon its important role in the NHEJ repair pathway, different anti-DNA-PKcs strategies have been explored to enhance sensitivity to radiation or DNA damage-based agents. A series of small-molecule ATP-competitive inhibitors of DNA-PKcs have been developed. Among these, NU7441 is the most potent and specific inhibitor of DNA-PKcs with a half maximal inhibitory concentration (IC_50_) of 14 nM against DNA-PKcs relative to other members of the PI3KK family (ATM and ATR) in a cell-free system [12]. Several studies have shown that NU7441 has potential value to enhance the radiosensitivity in different tumors, including colon cancer [13], breast cancer [14] and prostate cancer (PCa) [15]. Our previous data showed that NU7441 dramatically increased radiation sensitivity of highly aggressive and radiation resistant PCa cell lines [16].

In addition to inhibiting DSB repair, disturbing cell cycle checkpoint regulation, and inducing apoptosis, DNA-PKcs inhibition also promotes IR-induced cell killing through nonapoptotic responses, including mitotic catastrophe [17], senescence [18] and autophagic cell death [19]. Recent studies have shown that DNA-PKcs inhibits IR-induced mitotic catastrophe via promoting Chk2 activation, and consequently, that depletion of DNA-PKcs results in increased polyploidy and multipolar spindles after irradiation [17]. Azad et al demonstrated that inhibition of DNA-PKcs induces an accelerated senescence phenotype in irradiated human NSCLC [18]. Furthermore, depletion of DNA-PKcs radiosensitizes glioma-initiating cells due to IR-induced autophagic cell death which indicates DNA-PKcs involvement in autophagy progression [19]. We previously reported that inhibition of DNA-PKcs by NU7441 dramatically increased IR-induced autophagy in PCa cells [16]. However, how DNA-PKcs coordinates these different types of cell death pathways has not been well studied. Herein, to elucidate the cellular and molecular outcomes of inhibiting DNA-PKcs by NU7441 in irradiated NSCLC we studied the induction of apoptosis, mitotic catastrophe, autophagy and senescence in H460, A549 and H1299 cells. Our studies showed that NU7441 specifically radiosensitizes H460, A549 and H1299 cells through senescence, autophagy and mitotic catastrophe, respectively, and that the cell death pathway choice relies on the genotypic background of the tumor itself.

## RESULTS

### NU7441 sensitizes NSCLC cells to irradiation

**Figure 1:**
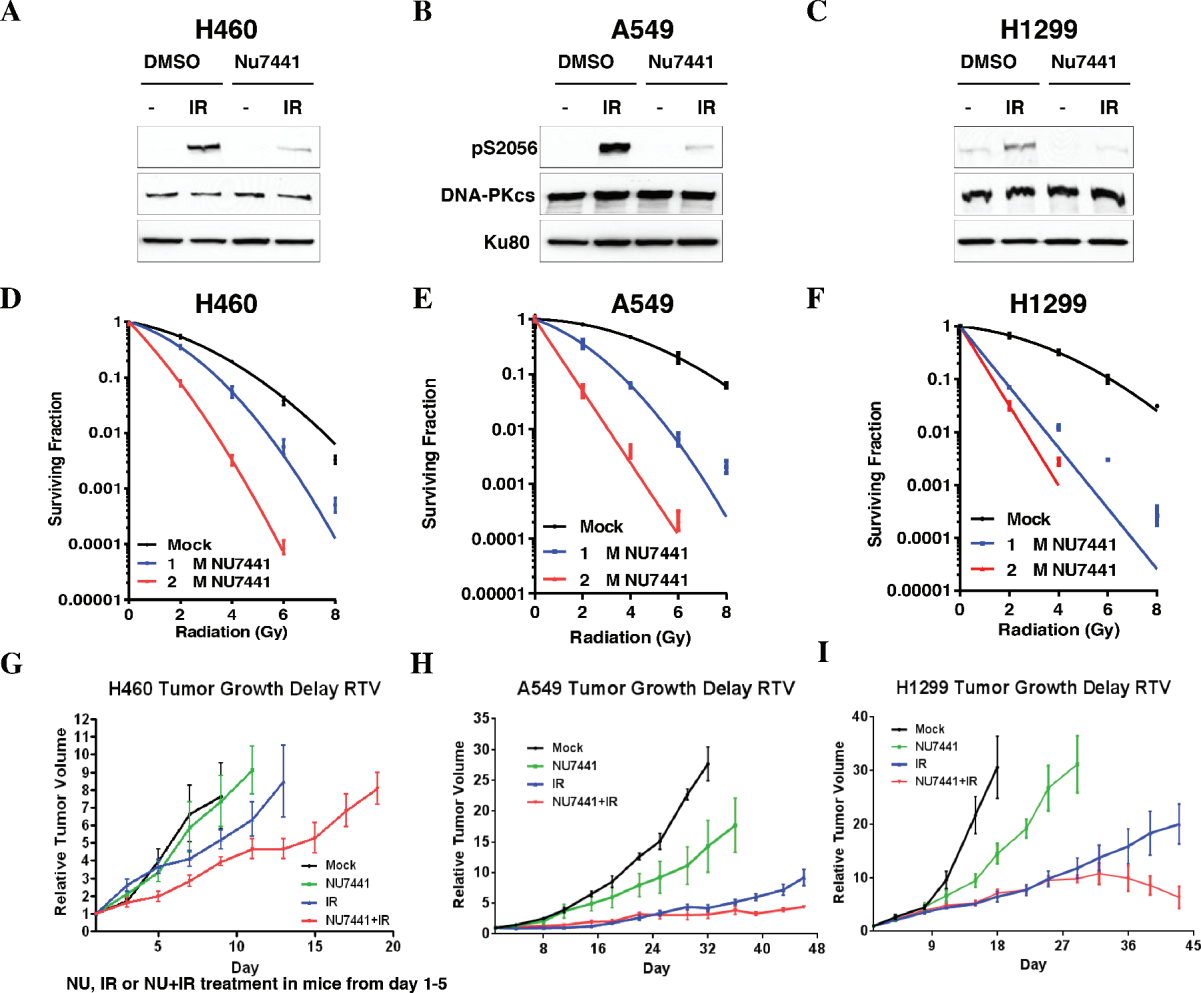
NU7441 sensitizes NSCLC cells to irradiation **(A–C)** H460, A549 and H1299 cells were irradiated (10 Gy, 1 hour) with or without pretreatment with NU7441 (10 μM), autophosphorylation of DNA-PKcs was determined. **(D–E)** Clonogenic survival of NSCLC cells with or without NU7441 H460, A549, and H1299 cells were treated with NU7441 (1 and 2 μm, respectively) for 1 hour and treated with IR as indicated. Cells were trypsinized immediately and counted and colony formation was performed. **(G–I)** NU7441 in combination with IR led to significant tumor growth delay in H460, A549 and H1299 cells.

To determine the effect of NU7441 on the radiosensitivity of NSCLC cells H460, A549 and H1299 cells were exposed to IR or NU7441 + IR. As shown in Figure 1A–1C, NU7441 can inhibit the IR-induced DNA-PKcs phosphorylation at its Ser2056 site which represents DNA-PKcs activation, but has no impact on the protein amount in all of these three cell lines. Using colony survival assay, we found that NU7441 significantly radiosensitized NSCLC cells. The surviving fraction (SF) at 2Gy (SF_2_) for H460, A549 and H1299 cells was reduced from 0.54 ± 0.04, 0.80 ± 0.04 and 0.67 ± 0.10, respectively, to 0.35 ± 0.03, 0.35 ± 0.07 and 0.07 ± 0.002 when we exposed the cells to 1 μM NU7441, and to 0.08 ± 0.01, 0.05 ± 0.01 and 0.03 ± 0.01when we treated with 2 μM NU7441, suggesting the radiation sensitizing effect of NU7441 is dose dependent (Figure 1D, 1E and 1F). To further analyze the efficacy of NU7441 as a radiosensitizer *in vivo*, tumor growth delay assays were performed (Figure 1G–1I). All three cell lines were implanted subcutaneously in athymic nude mice and allowed to form tumors and then treated with NU7441, radiation (IR), or IR + NU7441 as mentioned in the methods. We used relative tumor volume (RTV) as a measurement of treatment efficacy. Table 1 describes the days needed to reach a specific RTV for each tumor line. Growth delay (GD) was measured for each treatment to calculate a dose enhancement factor (DEF). We report a significant DEF of 1.7 and 1.6 in H460 and H1299 tumors respectively, whereas a modest DEF of 1.3 was observed in A549 tumors.

**Table 1:** The tumor growth delay in A549, H460 and H1299 cell lines

Tumors	RTV	Control (days)	NU7441 (days)	IR (days)	IR + NU7441 (days)	GD_Nu7441_ (days)	GD_IR_ (days)	GD_IR + NU7441_ (days)	DEF
**A549**	**5**	**12**	**16**	**35**	**46**	**4**	**23**	**34**	**1.3**
**H460**	**7**	**7**	**8.5**	**12**	**17**	**1.5**	**5**	**10**	**1.7**
**H1299**	**9**	**11**	**15.5**	**24.5**	**37.5**	**4.5**	**13.5**	**26.5**	**1.6**

**GD:** Growth Delay; **DEF:** Dose Enhancement Factor: (GD_IR + NU7441_-GD_NU7441_)/GD_IR_

### NU7441 blocks IR-induced DNA DSB repair in NSCLC

**Figure 2:**
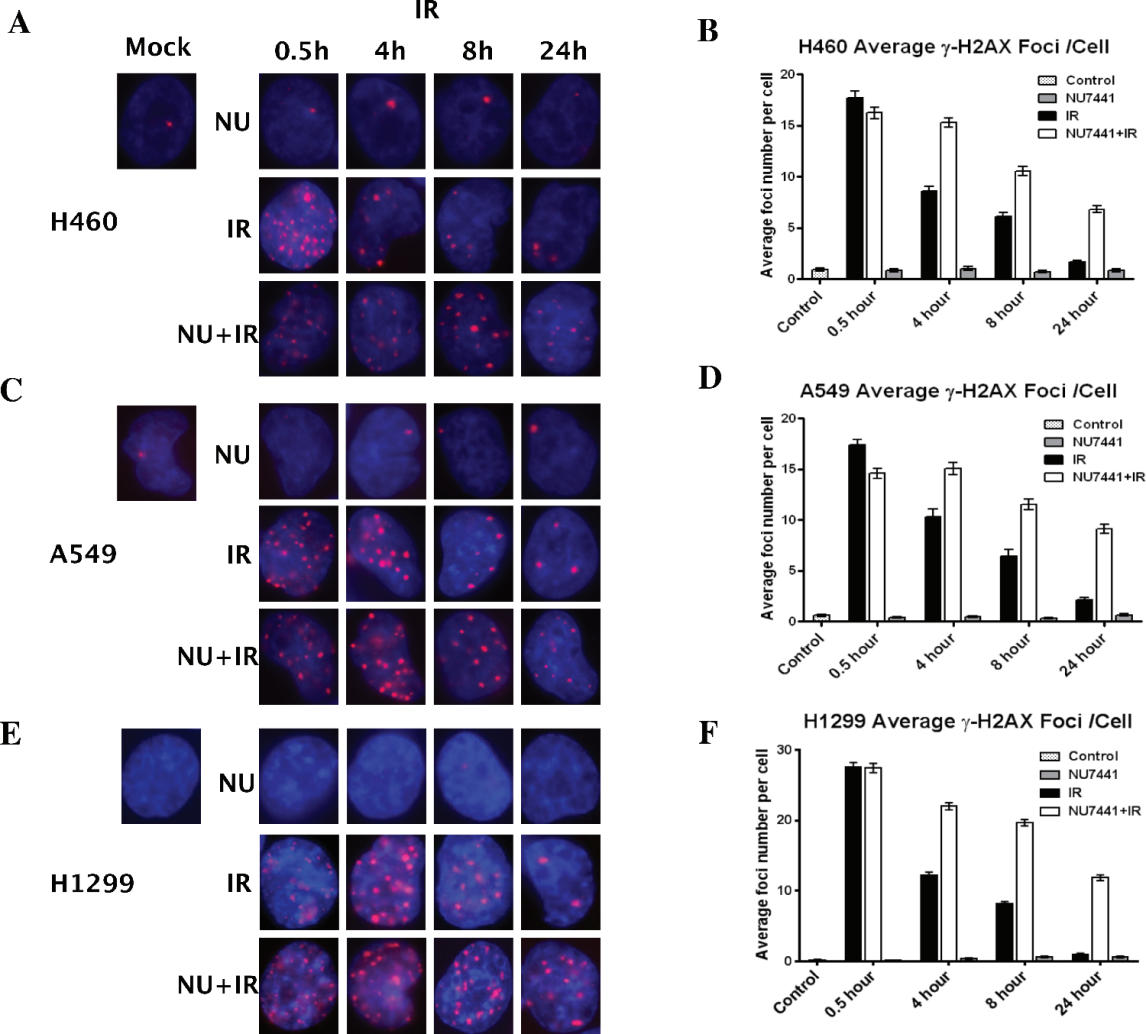
NU7441 enhanced cells sensitivity to IR correlates with deficient DSB repair NSCLC cells were irradiated with 2 Gy with or without NU7441 (1 hour prior to IR) and samples were collected at the indicated time points after IR, immunostained for phospo-γH2AX (red) foci and counted (average, 50 nuclei). **(A, C and E)** The representative image of H460, A549 and H1299 cells. **(B, D and F)** Quantitative analysis of DNA repair kinetics in NSCLC cells.

The rate of DNA DSB formation and subsequent rate of repair largely determine the efficacy of radiation therapy. To determine whether the increased radiosensitivity of cell lines after treatment with NU7441 was a product of compromised break repair, we subjected the cells to immunofluorescence staining for γH2AX (red). As shown in Figure 2A, 2C and 2E, IR-induced DNA damage could be observed within 30 minutes in all cell lines. In IR-only treated cells, most DNA DSB foci were repaired at 24 hours, whereas a dramatic number of γH2AX foci remained at 24 hours in NU7441 + IR treated cells (Figure 2B, 2D and 2F).

### NU7441 prolongs G2/M arrest in NSCLC but specifically leads to checkpoint abrogation and mitotic catastrophe in H1299 cells

**Figure 3:**
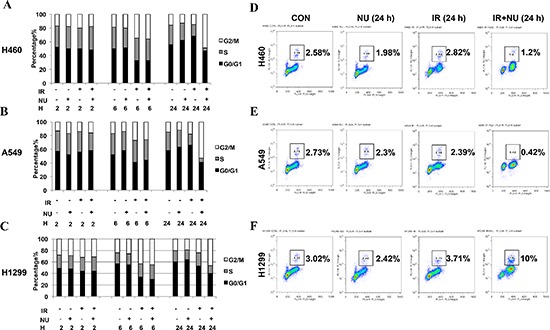
NU7441 treatment results in a robust G2/M cell arrest in NSCLC cells and specifically leads to checkpoint adaptation in H1299 cells **(A–C)** NSCLC cells were treated with IR (2 Gy), NU7441 (2 μM), and IR + NU7441 as indicated. Samples were collected at 0, 2, 6, and 24 hours post-treatment. Propidium iodide (PI) staining was used to detect the distribution of cells after various treatments. **(D–F)** The mitotic index was measured by flow cytometric analysis using PI staining for DNA content and anti–phospho-Histone H3 antibodies. Cells were irradiated at 2 Gy and samples were collected 24 h postradiation. Inset, the % of mitosis cells.

In response to IR, mammalian cells activate the cell cycle checkpoint which helps prevent cell division and provides necessary time for DNA damage repair. The effect of NU7441 on cell cycle distribution was analyzed by flow cytometry. As shown in Figure 3A, 3B and 3C, NU7441 induced a robust G2/M arrest from 6 to 24 hours after IR treatment in all cell lines. Moreover, the percentage of S phase cells was significantly decreased in H460 and A549, whereas H1299 cells had a relatively higher proportion S phase cells, suggesting H1299 cells may have a loose cell cycle checkpoint control system. To further distinguish mitotic cells from the G2/M group, flow cytometric analysis of histone H3 phosphorylation was performed. We found that NU7441 blocks H460 and A549 cells from entering M phase 24 hours after IR treatment. Interestingly, NU7441 treatment significantly increased mitotic arrest in H1299 cells (10%) (Figure 3D, 3E and 3F). As H1299 had the most DNA DSBs when compared to H460 and A549 cells, we speculate that NU7441 abrogates G2 arrest and enhances the DNA-damaging effects of radiation in H1299 cells.

**Figure 4:**
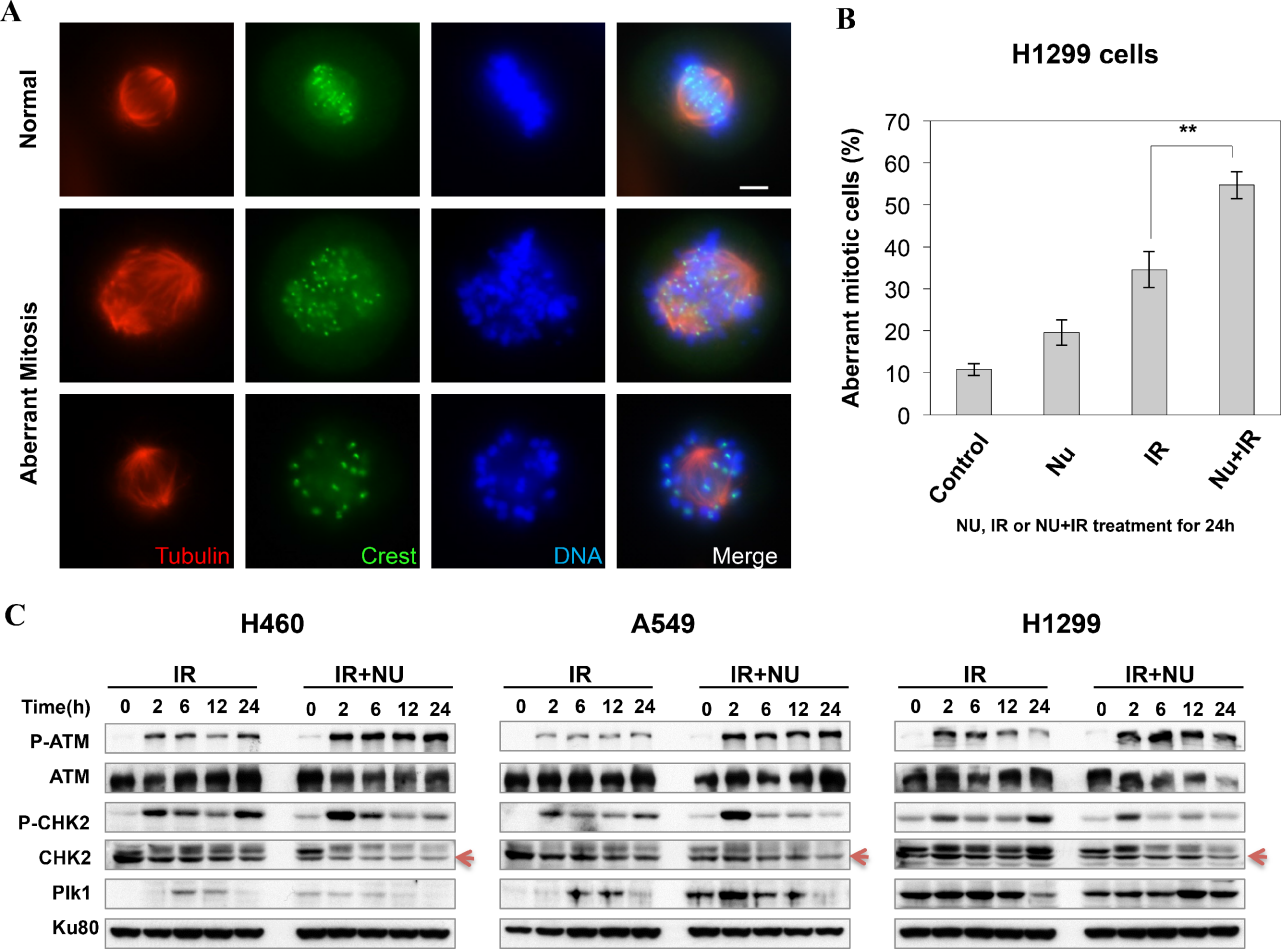
NU7441 + IR-induces mitotic catastrophe in H1299 cells **(A)** H1299 cells were stained with anti-α-tubulin and anti-crest antibodies, the representative images show the normal and aberrant mitosis in H1299 cells. **(B)** The percentage of aberrant mitotic cells is determined by the morphology of the spindle formation 24 h after NU7441 and IR treatment. As shown in the figure the number of aberrant mitosis increased after NU7441 and IR treatment. **(C)** The phosphorylation of ATM and Chk2 and the expression of Plk1 were determined by Western blot analysis at the indicated time points.

If cells escape from G2 arrest without completion of DNA repair and enter mitosis, cells likely will undergo mitotic arrest and then mitotic catastrophe (MC). Therefore, we then investigated mitotic catastrophe in NU7441 treated H1299 cells. As shown in Figure 4A and 4B, the structure of mitotic spindles was visualized by staining with antibodies against α-tubulin and CREST. Misaligned chromosomes and asymmetrical multipolar spindles were dramatically increased in NU7441 treated cells 24 hours after irradiation. This data supports the idea that NU7441 can specifically radiosensitize H1299 cells to IR through MC.

**Figure 5:**
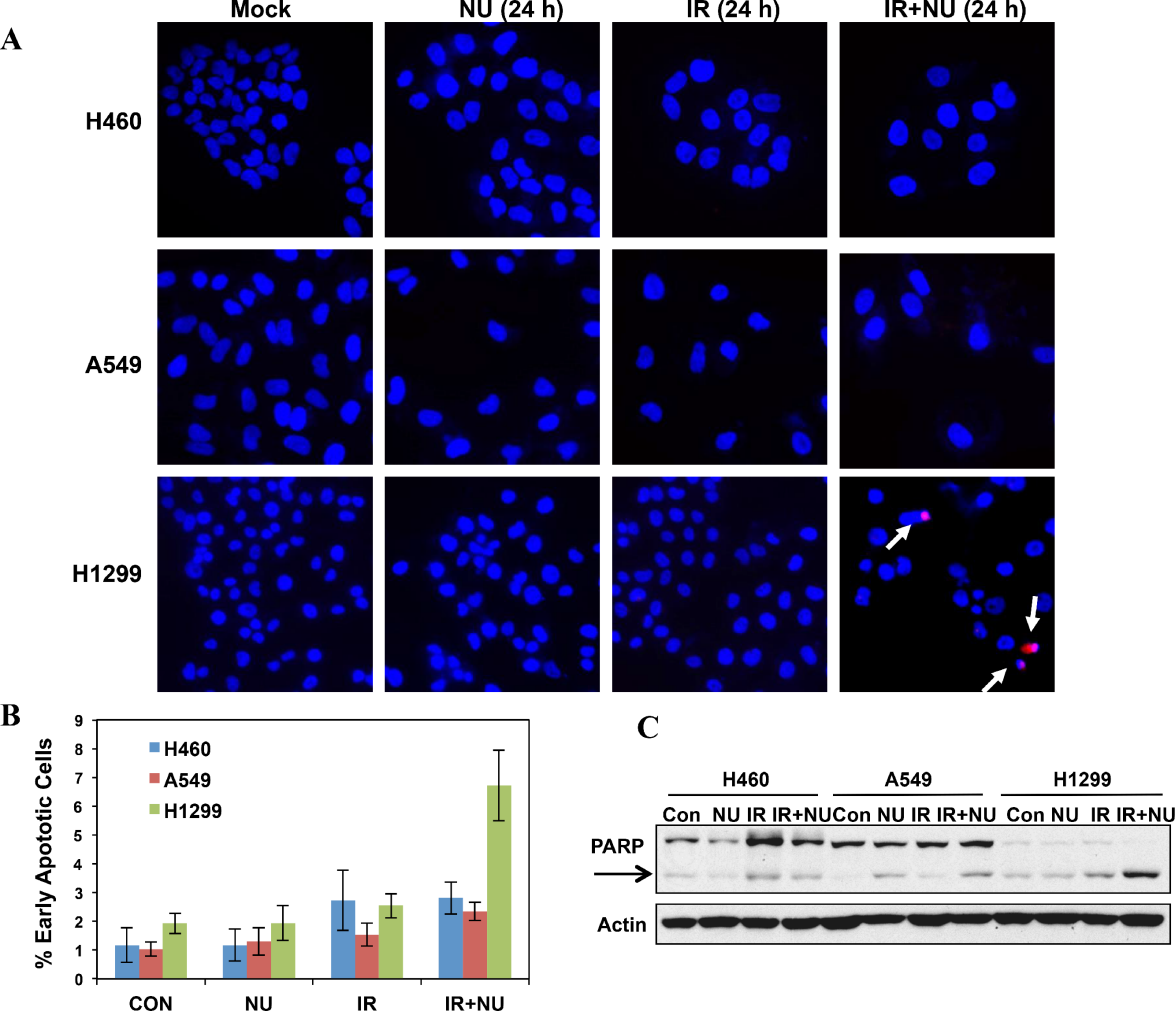
Mitotic catastrophe-related apoptosis was dramatically increased in NU7441 + IR treatment in H1299 cells **(A)** IR + NU7441-induced apoptosis was determined by cleaved-caspase 3 staining. Cells were treated with +/− NU7441 (5 μM) and IR (5 Gy) for 24 hours and the cells were stained with cleaved-caspase 3 and DAPI; the representative fluorescence images are shown. White arrows indicated the cleaved-caspase 3 positive apoptotic cells. **(B)** Quantitative analysis of apoptotic cells. The data are presented as the means ± SD of three independent experiments. **(C)** Analysis of PARP cleavage. Cells were lysed 24 hours after exposure to IR or IR + NU7441 and subjected to Western blot analysis.

p53 can prevent cells from going into cell cycle progression with damaged DNA. However, siRNA-mediated p53 knockdown A549 cells didn't lead to G2 checkpoint abrogation, suggesting p53 deficiency in H1299 cells is not essential for NU7441 induced G2 checkpoint abrogation in this cell line (data not shown). The ATM-Chk2 signal pathway plays an essential role in DSB or IR-induced G2 checkpoint activation. We found that IR-induced phosphorylation of ATM S1981 was increased in NU7441-treated cells indicating NU7441 treatment leads to more severe DNA damage, which is consistent with the DNA DSB repair kinetics data in Figure 2. Interestingly, ATM mediated Chk2 phosphorylation at T68 (Chk2 pT68) has peaks at 2 and 24 hours after IR treatment. As shown in Figure 4C, the T68-phosphorylated form of Chk2 at 24 hours is remarkably reduced in NU7441-treated H460, A549 and H1299 cells. In contrast, the Chk2 pT68 is dramatically increased in NU7441-treated H460 and A549, but not H1299 cells at 2 hours. Moreover, our study reveals that Plk1, which is involved in the process of IR-induced G2 checkpoint adaptation [20], is overexpressed in H1299 cells. Based upon these results, we hypothesize that Plk1 overexpression impairs the IR-induced G2 checkpoint and then leads to mitotic catastrophe in cancer cells in the presence of NU7441. Mitotic catastrophe is strongly associated with apoptosis. We further analyzed IR- and NU7441-induced apoptosis in these three cell lines. Cells were fixed and stained with cleaved-caspase3 (red) and DAPI (blue) 24 hours after NU7441 and/or IR treatment. As shown in Figure 5A, significant apoptosis (cleaved-caspase 3 positive) can only be observed in H1299 cells after combined treatment of IR + NU7441 after 24 hours (6.7%) (Figure 5B), which is consistent with its high level of mitotic catastrophe. To further characterize apoptotic events, a Western blot was performed to detect cleaved PARP-1 (Figure 5C). We noticed that PARP-1 cleavage is greater (lane 12) in H1299 cells compared to H460 and A549 cells 24 hours after IR + NU7441 exposure. These results support mitotic catastrophe as the predominant cause of apoptosis in H1299 cells.

### NU7441 treatment accelerates autophagy in irradiated NSCLC

**Figure 6:**
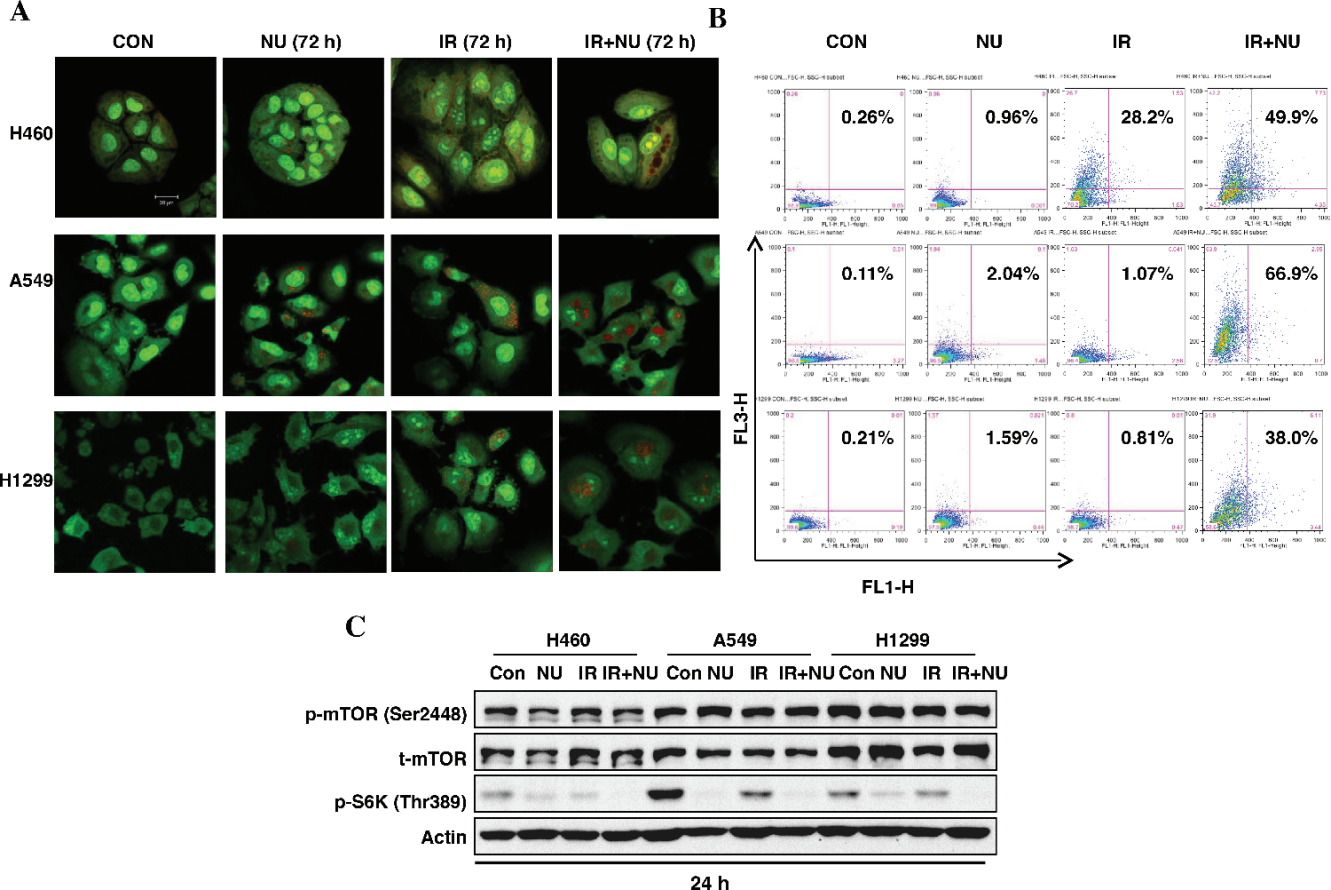
NU7441 + IR-induces autophagic cell death in NSCLC cells H460, A549 and H1299 cells were treated with NU744, IR, and IR + NU7441 for 72 hours and then stained with AO. **(A)** Fluorescent images of AVO-positive cells. **(B)** Flow cytometry analysis to assess autophagy. **(C)** Phosphorylation of mTOR and p70S6K were detected by Western blot analysis 24 hours after treatment.

Continuously accumulating data has suggested that radiation induces autophagy in a variety of cancer cells independent of apoptosis [21, 22]. Our previous study showed that NU7441 promotes radiation-induced autophagy in prostate cancer cells. Here we try to compare the autophagy responses in H460, A549 and H1299 cells when treated with IR or/and NU7441. The formation of acidic vesicular organelles (AVOs) is one of the characteristics of autophagy. Therefore, we analyzed the formation of AVOs using AO staining with flow cytometry. As shown in Figure 6A and 6B, little or no significant autophagy events were noticed after IR or NU7441 treatment alone in A549 and H1299 cells, only H460 cells showed significant autophagy after irradiation. However, all three cell lines exhibited increased autophagy with combined treatment of IR + NU7441. Moreover, A549 cells showed significant autophagy (66.9%) when exposed to IR + NU7441 for 72 hours, whereas H460 and H1299 cells showed only 49.9% and 38.0% at the same time point (Figure 6B). To further explore the role of NU7441 in the autophagy response, we looked at the activation of the mTOR-S6K signal pathway, which is strongly correlated with cellular autophagy. Interestingly, neither NU7441 alone nor NU7441 plus radiation affected phosphorylation of mTOR; however, IR + NU7441 treatment significantly inhibited phosphorylation of S6K (Figure 6C), indicating there are some mTOR-independent S6K mechanisms involved in NU7441-related autophagy regulation. Consistent with AVO staining results, A549 cells showed the strongest expression of phosphorylated S6K compared with H460 and H1299, and NU7441 almost totally inhibited S6K phosphorylation. These results suggest that S6K overactivated cells may exhibit more sensitivity to an autophagy inducer, such as NU7441.

### NU7441 specifically promotes senescence in irradiated H460 cells

**Figure 7:**
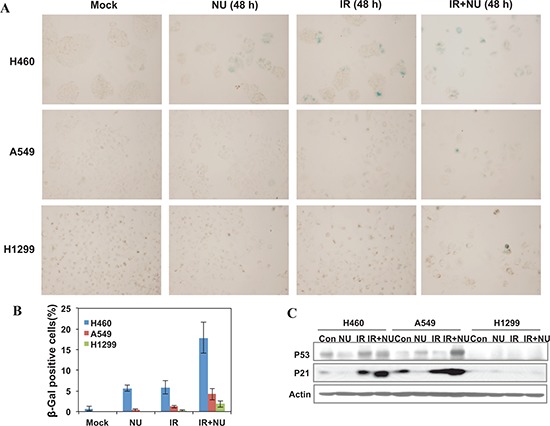
NU7441 enhanced IR-induced senescence in NSCLC cells H460, A549 and H1299 cells were treated with NU744, IR, and IR + NU7441 for 48 hours and then stained with X-Gal. **(A)** Representative image of SA-βGal activity after 48 hours treatment. **(B)** Quantitative analysis of senescent cells. The data are presented as the means ± SD of three independent experiments. **(C)** Cells were lysed 48 hours after exposure to IR or IR + NU7441 and subjected to Western blot analysis, the expression of p53 and p21 were determined.

Recent work has shown that treatment with BEZ235, a double inhibitor of DNA-PKcs and mTOR, radiosensitizes A549 and H460 cells through accelerated senescence. Hence, we investigated the effect of NU7441 on the IR-induced senescence phenotype in all of these cell lines. Senescence-associated beta-galactosidase (SA-β-gal) activity was examined using SA-β-gal staining. As shown in Figure 7A–7B, NU7441 + IR treatment leads to a marked increase of senescence in H460 cells (17.8%) compared with A549 (4.2%) and H1299 (1.8%) cells. Because the p53-p21 pathway plays an essential role in DNA damage-induced senescence, we therefore measured the protein expression of this signal pathway and found that IR activates the p53-p21 pathway in both A549 and H460 cells but not in p53-deficient H1299 cells, and that NU7441 treatment further promotes p53 and p21 accumulation in A549 and H460 cells (Figure 7C). Several studies have revealed that p53-p21 also plays a pivotal role in autophagy, however the mechanism by which p53-p21 regulates the pathway choice between autophagy and senescence is still an unanswered question that is currently under investigation.

## DISCUSSION

Radiotherapy is an effective strategy for the treatment of non-small cell lung cancer but several lung cancer cell lines display significant radiation resistance that limits the success of radiotherapy in advanced lung cancer [23]. Therefore, it is imperative to develop novel treatment strategies to enhance radiotherapy efficacy and improve the prognosis of NSCLC. Here, we investigated the therapeutic potential of NU7441, a novel specific inhibitor of DNA-PKcs, in H460 (K-Ras mutation), A549 (K-Ras mutation) and H1299 (N-Ras mutation and p53 null) NSCLC cells. We observed significantly enhanced cell killing in all NSCLC cells when exposed to the combination of radiation and NU7441 both *in vitro* and *in vivo*. DNA-PKcs is activated upon recruitment and association with the Ku70/80 heterodimer at DSB sites, and that kinase activity is essential for the NHEJ pathway, the predominant mechanism to repair IR-induced DSBs in mammals [24]. It was reported previously that the expression of a kinase-dead mutant DNA-PKcs results in severe defects in DSB repair and clonogenic survival against IR [25]. Although the requirement of DNA-PKcs kinase for the NHEJ pathway is not fully elucidated, DNA-PKcs is known to phosphorylate many downstream effectors of the NHEJ pathway as well as additional factors continuously being identified [24]. The development of various DNA-PKcs kinase inhibitors and their efficacy in blocking DSB repair further highlights the critical function of DNA-PKcs and indicates a therapeutic target for improving radiotherapy in cancer treatment [10].

Recently, several studies revealed that inhibition of DNA-PKcs promotes IR-induced cell killing via multiple cell death pathways, including programmed cell death [7, 8], mitotic catastrophe [17], autophagy [19] and senescence [18]. To clarify how DNA-PKcs coordinates different cell death pathways after irradiation, we analyzed these cell death responses in H460, A549 and H1299 NSCLC cell lines when treated with NU7441 and/or IR. Mitotic catastrophe is the main mode of cell death following treatment with ionizing radiation and is defined as an aberrant form of mitosis associated with prolonged mitotic arrest and various morphological changes [26, 27].

Consistent with previous reports, treatment with NU7441 resulted in robust G2/M arrest 24 hours after irradiation in all three NSCLC cell lines used in this study. Interestingly, when we further separated G2 and M phases using anti-pH3-Ser10 antibodies, mitotic arrest was only found in H1299 cells after treatment with NU7441 (Figure 3).

The G2 checkpoint stops cells from progressing into mitosis and allows the DNA repair machinery time to fix lesions. However, when the G2 checkpoint is weakened cells cannot maintain prolonged cell cycle arrest and enter mitosis before DNA damage is repaired [28]. The premature entry into mitosis with unrepaired DNA disturbs the mitotic kinetochore-microtubule structure and leads to mitotic arrest due to spindle checkpoint activation [29]. We found a higher incidence of aberrant mitotic spindle structures after combined treatment with NU7441 and IR in H1299 cells. Altogether, our data demonstrates that NU7441 treatment leads to increased mitotic catastrophe specifically in H1299 cells, indicating dysregulation of the G2 checkpoint in H1299 cells, either due to weakened checkpoint machinery or enhanced checkpoint recovery due to Plk1 overexpression. Chk2 is an important kinase involved in G2 checkpoint maintenance [30]. In response to irradiation, ATM phosphorylates Chk2 at its Thr68 residue promoting Chk2 dimerization and full activation [30]. Additionally, Chk2 is also a target of DNA-PKcs after irradiation and DNA-PKcs-Chk2 activation inhibits IR-induced mitotic catastrophe [17]. Recently, several studies demonstrated that phosphorylation of Chk2 T68 participates in mitotic spindle assembly and microtubule organization, and that DNA-PKcs is responsible for mitotic Chk2 T68 phosphorylation [31]. Here, we found that the phosphorylation of Chk2 at Thr68 is more robust 2 hours after irradiation in NU7441-treated H460 and A549 cells when compared to IR alone. However, NU7441 treatment significantly inhibits Chk2 pT68 at a later time point after irradiation in all three of these cell lines. Based upon this data, we speculate that the activation of Chk2 at 2 hours after IR represents its function at the G2 checkpoint, whereas, the phosphorylation of Chk2 at later time points correlates with its role in mitosis regulation, which is dependent on the DNA-PKcs kinase and is attenuated in the presence of NU7441. Plk1 is an essential kinase for mitotic progression [32, 33] that is overexpressed in many cancers, including NSCLC [34]. Besides its function in normal mitotic progression, Plk1 plays a crucial role in DNA damage-induced G2 checkpoint recovery [35]. Activation of Plk1 is correlated with G2 checkpoint abrogation [20]. Van Vugt et al demonstrated that Plk1 blocks Chk2 activation even in the presence of active ATM during mitosis [36]. In this study, we identified that Plk1 is overexpressed in H1299 cells compared with H460 and A549, indicating that Plk1 may partially impair radiation-induced G2 checkpoint via disturbing Chk2 phosphorylation in H1299 cells. Overexpression of Plk1 may further sensitize cells to combined NU7441 and IR treatment through mitotic catastrophe. We speculate that the expression level of Plk1 might play an important role in determining the choice between mitotic catastrophe and other cell death pathways. Recent studies support the idea that mitotic catastrophe is a subtype of apoptosis induced by a combination of defective cell cycle checkpoints and persistent DNA damage [37, 38]. Consistent with these reports, our study revealed that NU7441 treatment significantly enhanced IR-induced apoptosis in H1299 cells, whereas only a very low percentage of apoptotic cells were detected in the other two cell lines.

Autophagy is a lysosomal degradation pathway that eliminates damage or potentially dangerous proteins and organelles under adverse conditions to protect cells from metabolic stress [39]. However, previous studies have suggested that autophagy also functions as a pro-death mechanism that is frequently activated in tumor cells treated with chemotherapy or radiotherapy [40, 41]. Previous studies have demonstrated that DNA-PKcs activation is involved in the cellular decision to undergo apoptosis or autophagy. Loss or inactivation of DNA-PKcs causes autophagic cell death in malignant gliomas after low-dose irradiation [19]. We found that combined treatment with NU7441 + IR dramatically induced autophagy in NSCLC cells especially in A549 cells (66.9%). Western blot analysis showed that DNA-PKcs suppression markedly decreased the phosphorylation of p70S6K at its Thr387 site but did not affect mTOR phosphorylation. The p70S6k-mediated phosphorylation of S6 protein, a component of eukaryotic ribosomal 40S subunit, is important for protein translation and ribosome formation [42]. On the basis of the experiments with rat hepatocytes, p70S6K is a negative regulator of autophagy [43, 44]. Here, we found that the phosphorylation level of p70S6K is dramatically higher in A549 cells compared to H460 and H1299. Therefore, we speculate that phosphorylation of p706K may be a molecular marker to predict sensitivity of autophagy induction. More interestingly, other mTOR-independent pathways must exist in order to explain the phenomenon that DNA-PKcs inhibition has no effect on mTOR phosphorylation. It has been reported that phosphoinositide-dependent kinase (PDK1) can phosphorylate and activate p70 S6K [44, 45]. Moreover, the PDK1 signal pathway is correlated with DNA-PKcs activation after irradiation through coordinating PKB/Akt phosphorylation [46]. The crosstalk between PDK1 and DNA-PKcs with respect to IR-related autophagy will require further investigation.

Direct DNA damage, caused by either radiation or DNA-damage agents, can induce cell senescence [47]. Defects in DSB repair have been correlated to senescence phenotype. Knockout of Xrcc4, DNA ligase IV, Ku86, Brca1 and other DNA repair-related genes induces premature senescence of MEFs and results in an aging phenotype in mice [48]. In this study, we revealed that NU7441 can significantly accelerate IR-induced cellular senescence in H460 cells but only little senescence was observed in A549 and H1299 cells. The p53-p21 signal pathway plays a pivotal role in mediating DNA damage-induced senescence. Hence, H1299 has little senescence because of its p53-deficient status. However, IR and IR + NU7441 combined treatment leads to increased expression of p53 and p21 in both H460 and A549 cells. It is not clear how cells choose between cell senescence (H460) and autophagy (A549) in response to DNA damage. Even though the protein amount was similar between A549 and H460 cells, we cannot exclude the possibility that different post-translational modifications of p53 and its different partners might exist in these two cell lines which could lead to the activation of different sets of transcriptional targets and subsequently determine cell death pathway choice. Several recent studies demonstrated that autophagy and senescence tend to occur in parallel and that autophagy enhances the senescent phenotype [49, 50]. On the basis of these reports, we hypothesized that autophagy might also be a primary molecular event and a driver of senescence after IR + NU7441 treatment. The phenotypes of senescence and autophagy that have been observed in H460 and A549 cells respectively may represent different stages of cell death progression.

We also looked at the possible lung toxicity that may occur during the combined treatment of radiation and NU7441 in animal models. Since athymic nude mice are deficient in immune response, we used an immune competent mouse strain (C3H/HeJCr). For this study we used a single fraction of 6 Gy or 10 Gy +/− NU7441 targeted to the left lung using an image guided irradiator (X-Rad 320) with a 3.5 mm collimator. After 60 days, lungs were fixed by tracheal instillation of 10% neutral buffered formalin and histopathologic examinations were performed using H & E staining. We did not observe any pneumonitis at these time points after treatment with radiation and NU7441. We noticed minor fibrosis only after 60 days in mice treated with s single fraction of 10 Gy, 6 Gy + NU7441 and 10 Gy + NU7441 as indicated in the figure (Supplementary Figure 1).

In conclusion, this preclinical study clearly shows that NU7441 can be administered with IR to improve the efficacy of radiation therapy in NSCLC cells. More importantly, our work has demonstrated that NU7441 + IR treatment can induce different cell death events, including mitotic catastrophe, apoptosis, autophagy and senescence. Lastly, and most interestingly, cell death pathway choice may be dependent on the genetic milieu of cancer cells implying that NU7441 can be an important part of a personalized treatment strategy.

## MATERIALS AND METHODS

### Cell culture and treatment

The human NSCLC cell lines H460, A549 and H1299 were kindly provided by Dr. John D. Minna at University of Texas Southwestern Medical Center, Dallas, TX, and grown in RPMI 1640 medium with 10% FBS (HyClone, Hudson, NH, USA) at 37°C with 5% CO_2_. Cells were treated with ionizing radiation using a Mark-II Cesium-137 irradiator (J L Shepherd and Associates) at a dose rate of 3.47 Gy/min at room temperature with or without a 1 hour pretreatment with NU7441 (Tocris Bioscience, Ellisville, Mo, USA).

### Immunoblotting and antibodies

Whole-cell lysate preparation and western blotting were performed as previously described [51]. For immunofluorescent staining, cells were grown on poly-D-lysine-coated culture slides (BD Pharmingen, San Diego, CA, USA), washed in phosphate-buffered saline (PBS), fixed in PBS containing 4% paraformaldehyde, permeabilized in 0.5% Triton X-100 and blocked in PBS containing 5% bovine serum albumin. The cells were incubated with indicated primary antibodies for 2 h at room temperature, washed with PBS and incubated with Alexa-568- and Alexa-488- conjugated secondary antibodies for 1 h (Invitrogen). Cells were then washed with PBS and mounted in Vectashield mounting medium with 4,6-diamidino-2-phenylindole (Vector Laboratories, Burlingame, CA, USA). Images were acquired from a Zeiss AxioImager M2 microscope system equipped with a Plan-Apochromat 63/NA 1.40 objective, an AxioCam MRm CCD camera and AxioVision software (Carl Zeiss, Oberkochen, Germany). Anti-phospho-histone γH2AX (Ser139) was purchased from EMD Millipore. Mammalian target of rapamycin (mTOR), phosphor-mTOR (pmTOR, S2448), phospho-S6 kinase (pS6K, T389), Plk1, ATM, phospho-ATM (pATM, S1981), Chk2, phosphor-Chk2 (pChk2, T68), p53, p21 and poly (ADP-ribose) polymerase (PARP) antibodies were purchased from Cell Signaling Technology (Danvers, MA). Anti-actin antibody was purchased from Sigma-Aldrich (St Louis, MO). Fluorescent dye-conjugated secondary antibodies were obtained from Invitrogen. Antibodies against total and phosphorylated forms of DNA-PKcs were described previously [4, 5].

### Clonogenic survival assay

Clonogenic survival experiments were performed as previously described [51].

### Cell cycle analysis

After washing twice with PBS solution, the treatment or control cells were collected and fixed using 75% ethanol at −20°C for at least 24 hours. The cells were resuspended with PBS and incubated with 20 μl 1 mg/ml RNase A (Sigma, St Louis, MO) for 30 min at 37°C, and stained with 25 μg ml/ml propidium iodide (Sigma) for 30 min at room temperature. The cell cycle distribution was determined using flow cytometry, and more than 10,000 cells per sample were counted.

### Detection of acidic vesicular organelles

The cells were grown in 6-well plates and allowed to attach overnight. The cells were incubated with 1 μg/ml acridine orange/PBS for 15 min, washed with PBS, and examined under a LSM 510 laser-scanning confocal microscope (Carl Zeiss, Oberkochen, Germany) at × 63 magnification 72 hours after irradiation or/and NU7441 treatment. Untreated cells were also cultured for 72 hours as a negative control. The samples were collected for FACScan and analyzed using Flowjo 8.7.1 (Tree Star, Inc, Ashland, OR) software to quantify cells that were positive for acidic vesicular organelles (AVOs).

### Senescence-associated β-galactosidase staining

Cells were treated with NU7441 or/and irradiation for 48 hours and then fixed in 2% fomaldehyde/0.2% glutaraldehyde for 5 min at room temperature. Rinse the fixed cells twice with PBS and add β-galactosidase staining solution containing 20 mg/mL X-gal (Promega), cells were incubated for 6–10 hours at 37°C incubator without CO_2_.

### Tumor growth delay

H460, A549 and H1299 NSCLC cells were injected subcutaneously (1 × 10^6^ cells in 100 μL) into the right posterior flanks of female athymic nude mice (nu/nu, 5–6 weeks old). Tumors were treated when they reached 5 to 6 mm in diameter. Treatment groups (5 animals per group) included untreated control (received 0.9% saline), those treated with NU7441 (25 mg/kg/day for 5 days, by IP), with radiation (2 Gy/day, 5 days), and those that received combined treatment with NU7441 and IR. NU7441 was administered 1 h before radiation. Tumor growth delay and the dose enhancement factor were then determined [51]. Relative Tumor Volume (RTV) was determined by the ratio of the tumor volume at the indicated day divided by the tumor volume on the 1st day of treatment prior to treatment. All experiments were conducted under Institutional Animal Care and Use Committee of UTSW approved guidelines for animal welfare. The data are presented as the means ± SEM.

### Statistical analysis

Data is presented as the mean ± SD of at least three independent experiments. The results were tested for significance using the unpaired Student's t test.

## SUPPLEMENTARY MATERIAL, FIGURE


