# Supplementary material for: NSCLC cells demonstrate differential mode of cell death in response to the combined treatment of radiation and a DNA-PKcs inhibitor
Source: Oncotarget. 2015 Feb 28;6(6):3848–60. doi: 10.18632/oncotarget.2975 (PMC4414158; doi:10.18632/oncotarget.2975)
Supplement: Supplementary file 1 [file oncotarget-06-3848-s001.pdf]

SUPPLEMENTARY MATERIAL

DETAILED METHODS FOR NORMAL TISSUE TOXICITY IN RESPONSE TO RADIATION AND NU7441 TREATMENT

For this study, immune competent mice strains (C3H/HeJCr) were used. Animals were treated with NU7441 (25mg/kg, I.P. single dose), radiation (single fraction of 6 and 10 Gy) and radiation (6 Gy, 10 Gy) +NU7441 (25mg/kg, I.P. single dose). For the combined treatment, NU7441 was administered 1 h prior to radiation treatment. A small animal irradiator (X-Rad 320) was used for radiation treatment into the left lung with image guidance using 3.5 mm collimator as described by Cho *et al* [1]. After 60 days, lungs were fixed by tracheal instillation of 10% neutral buffered formalin and histopathologic examinations were performed using H & E staining.

REFERENCE

1. Cho J, Kodym R, Seliounine S, Richardson JA, Solberg TD, Story MD. High dose-per-fraction irradiation of limited lung volumes using an image-guided, highly focused irradiator: simulating stereotactic body radiotherapy regimens in a small-animal model. Int J Radiat Oncol Biol Phys. 2010; 77:895–902.

SUPPLEMENTARY FIGURE

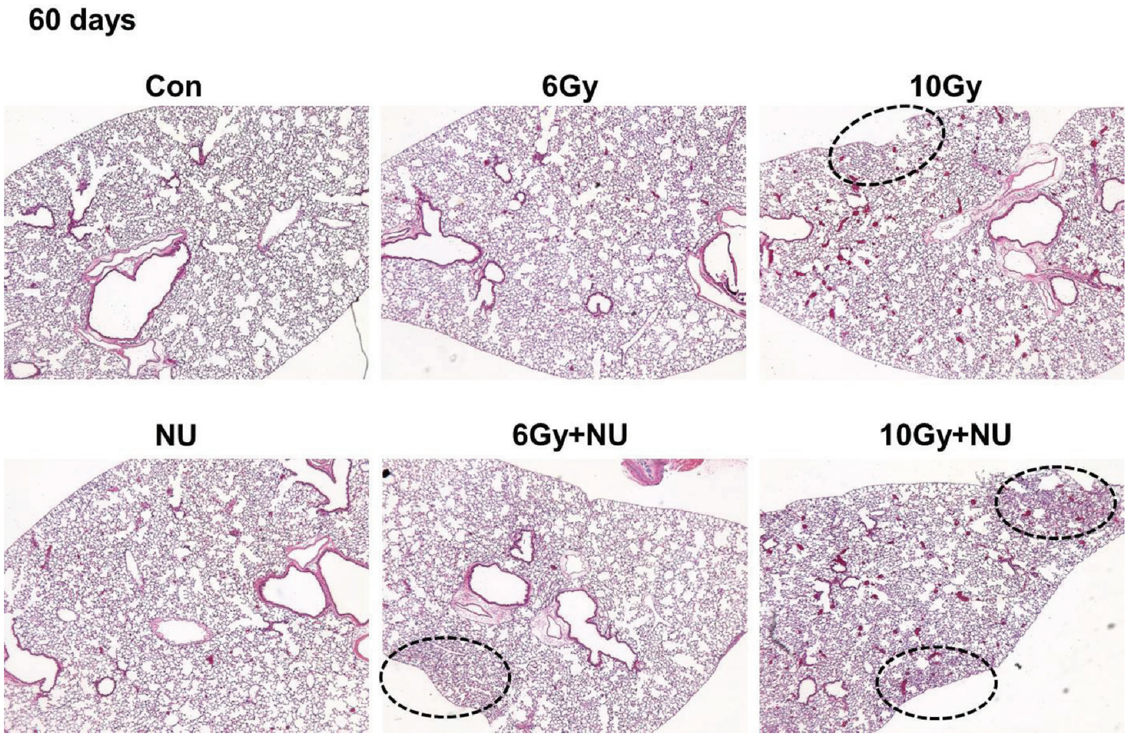

Supplementary Figure S1: NU7441 and radiation cause minimal increased normal tissue toxicity. C3H/HeJCr mice were irradiated with a single fraction of 6 or 10 Gy with or without NU7441 (25 mg/Kg) to the left lung. As shown in the Supplementary Figure 1, the addition of NU7441 only minimally increased parenchymal changes indicative of fibrosis in both the 6 Gy and 10 Gy groups (circled in black above) after 60 days of treatment.
